# Supplementary material for: High-Frequency Transcranial Random Noise Stimulation Modulates Gamma-Band EEG Source-Based Large-Scale Functional Network Connectivity in Patients with Schizophrenia: A Randomized, Double-Blind, Sham-Controlled Clinical Trial
Source: J Pers Med. 2022 Sep 30;12(10):1617. doi: 10.3390/jpm12101617 (PMC9605300; doi:10.3390/jpm12101617)
Supplement: Supplementary file 1 [file jpm-12-01617-s001.zip › Supplementary materials.pdf]

Supplementary materials

High-frequency transcranial random noise stimulation modulates gamma-band EEG source-based large-scale functional network connectivity in patients with schizophrenia: a ran-domized, double-blind, sham-controlled clinical trial

#Corresponding author: Hsin-An Chang, M.D. (Professor and Attending Psychiatrist)

Department of Psychiatry, Tri-Service General Hospital, No. 325, Cheng-Kung Road, Sec. 2, Nei-Hu District, Taipei, 114, Taiwan

Tel/Fax: 011-886-2-8792-7220 / 011-886-2-8792-7221

E-mail: chang.ha@mail.ndmctsgh.edu.tw

This supplementary information contains:

Supplementary text, figures, tables, and references

1. Supplementary text

Effectiveness of blinding

One hour after the 1st session of stimulation, all participants were asked to answer the question of whether they had received active hf-tRNS or sham stimulation. After unblinding of the study, it was shown that 76.5% of participants receiving active hf-tRNS and 72.2% of those receiving sham tRNS guessed they had received active stimulation. Fisher's exact test showed no statistically significant between-group differences ($p = 1.00$). Thus, the adequacy and effectiveness of our blinding protocol were considered satisfactory. Furthermore, the participants who predicted that they had received active stimulation did not have higher responder rates for negative symptoms shortly after 10-session stimulation (22.2% versus 19.2%, $p = 1.00$, Fisher's exact test) nor did they have greater decreases in PANSS-FSNS scores shortly after 10-session stimulation ($r = 0.00$, $p = 1.00$, point biserial correlation analysis), at the one-week ($r = -0.09$, $p = 0.61$) and one-month ($r = -0.07$, $p = 0.68$) follow-ups.

Dropout

Dropout was considered after the absence in two consecutive hf-tRNS sessions or declined consent to participate after receiving the first hf-tRNS session. In this trial, 36

patients were randomly allocated to receive active hf-tRNS (n=18) or to the sham (n=18) (See figure S1: CONSORT Flowchart) and 35 of them completed 10 sessions of stimulation. One participant in the active group received 4 sessions of stimulation and then dropped out due to a non-medical reason. Other participants had high adherence to the trial visits without any missing visit.

Online correction of eye movement and artifacts

The software module built in the Neuro Prax® TMS/tES compatible full band DC-EEG system provides correction of EEG artifacts in real time caused by blinking and eye or body movements. By choosing a correction mode of "artifact correction and correction of eye movements" from the box at the toolbar, the EEG signal was processed by a special algorithm [1] that has been developed by the manufacturer to (1) eliminate signal changes exceeding a certain threshold (standard value is 200 μV) indicating artifacts, e.g., movements of patient or cables during EEG recording, and (2) eliminate or suppress signals from the eye on the EEG without disturbing the EEG signal itself. Calibration was necessary to estimate the influence of horizontal movements, vertical movements and blink artifacts on the EEG. The calibration was done every time after applying electrodes on the patients' head. After carefully preparing the skin and applying the electrodes for EOG recording the calibration took approx. 3 minutes. An impedance check was carried out before calibration. The calibration consisted of 3 consecutive tasks. The participants

were asked to: (1) move the eyes in vertical direction with maximum deflection, (2) move the eyes in horizontal direction with maximum deflection, and (3) open and close the eye alternatively. After completing all tasks the calibration data were calculated. The data records on hard disk were the original data which were not influenced by the online correction. Corrected data were subsequently exported by means of the optional "Export Tool", e.g., to EDF+.

The analyses of whole-brain electrical source functional connectivity

In this study, whole-brain functional connectivity analyses were performed by the ‘‘whole-brain Brodmann areas (BAs)’’ approach, using the anatomical definitions of 84 BAs provided by eLORETA software package and based on the Talairach Daemon (http://www.talairach.org/). The standard electrode positions on the MNI152 scalp were used and the intracerebral volume's partitioning was restricted to 6239 voxels of 5 × 5 × 5 mm spatial resolution. The electric activity at each voxel in the neuroanatomic Montreal Neurological Institute (MNI) space as the exact magnitude of the estimated current density is represented by the eLORETA images. We used Brodmann areas as anatomical labels using MNI space with correction to Talairach space. We chose a ROI-maker 2 method (available in eLORETA) for the construction of the regions of interest (ROIs). Brain activity was computed with eLORETA in 42 regions positioning the center in Brodmann Areas (BAs: 1, 2, 3, 4, 5, 6, 7, 8, 9, 10, 11, 13, 17, 18, 19, 20,21, 22, 23, 24, 25, 27, 28, 29, 30, 31,

32, 33, 34, 35, 36, 37, 38, 39, 40,41, 42, 43, 44, 45, 46, 47) for each hemisphere, in order to get a topographic view. Pairs of BAs were analyzed using the values of single voxels with the highest F-ratio value at the centroid of each BA. Analyses of whole-brain functional connectivity (FC) were based on lagged phase synchronization (LPS) and were performed by use of all 42 BAs in each hemisphere as regions of interest (ROIs) to test interregional functional correlations between any pair of BAs LPS. FC values were obtained for all possible pairs of 84 ROI's, for each subject and for EEG frequency band of gamma (33–45 Hz). Based on normalized Fourier transforms, LPS algorithm evaluates the similarity between signals in the frequency domain. Hence, LPS is method associated with nonlinear functional connectivity and represents the connectivity of two signals after excluding the instantaneous zero-lag component. The results of the analyses of whole-brain electrical source functional connectivity can be seen in Table S3 and Figure S2.

2. Supplementary figures

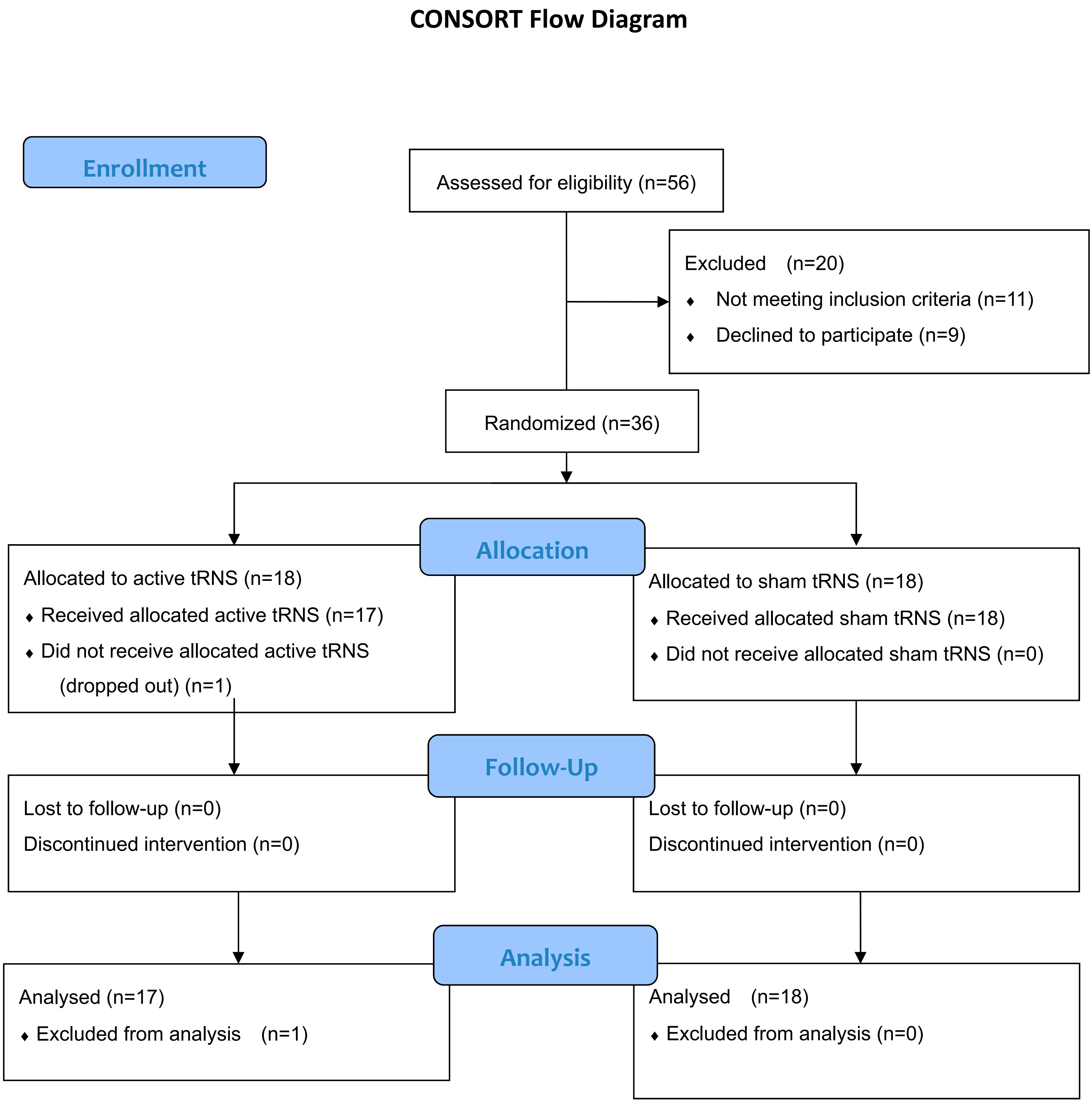

Figure S1. A CONSORT Flow Diagram of this clinical trial.

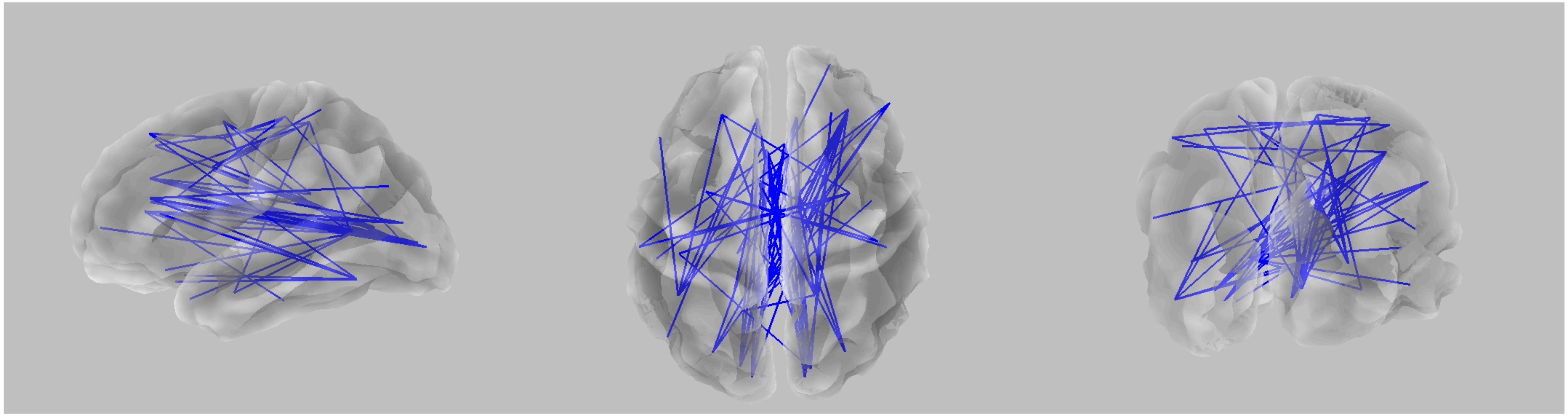

Figure S2. Functional connectivity analyses with whole-brain Brodmann areas (BAs) approach, using the anatomical definitions of 84 BAs provided by eLORETA software package. Relative to sham group, patients treated with hf-tRNS showed significantly reduced functional connectivity wires in the gamma frequency at the end of stimulation according to eLORETA maximal t-threshold ($t > 2.825$, p2-tailed < 0.05). From left to right: left sagittal, top, and coronal views. The blue color of the wire indicates functional connections with reductions in values of lagged phase synchronization (LPS) from baseline to the end of stimulation in patients treated with hf-tRNS compared to sham group. Please, refer to Table S3 in the Supplementary materials for statistical details of the changes in functional connectivity between 84 regions positioning the center in BAs.

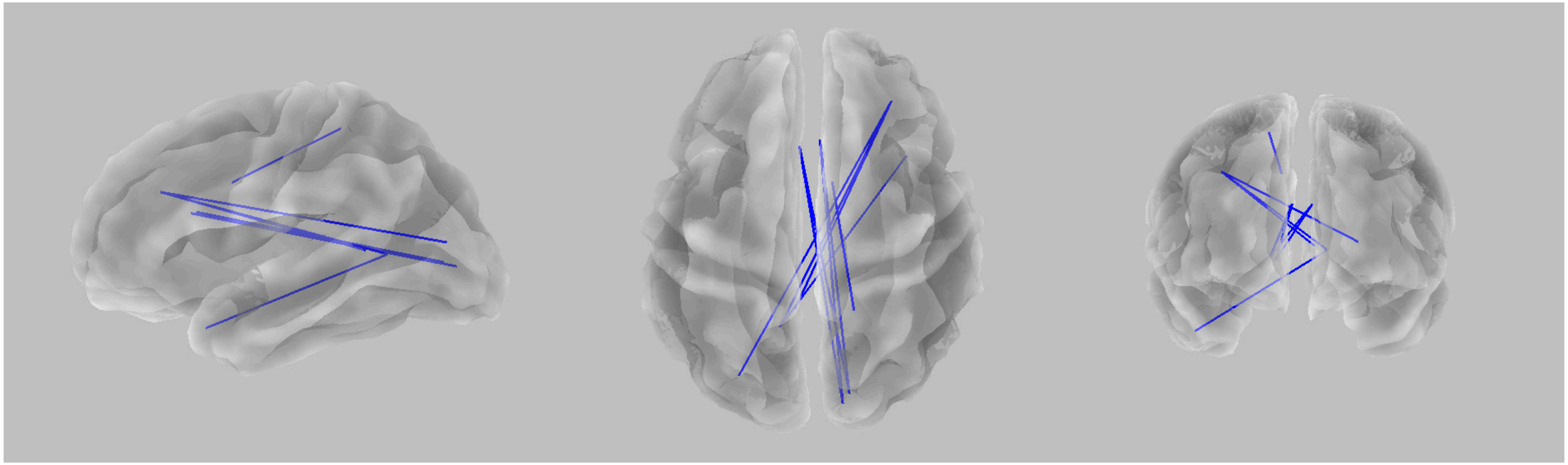

Figure S3. Functional connectivity analyses with whole-brain Brodmann areas (BAs) approach, using the anatomical definitions of 84 BAs provided by eLORETA software package. Relative to sham group, patients treated with hf-tRNS showed significantly reduced functional connectivity wires in the gamma frequency at the end of stimulation according to eLORETA maximal t-threshold (t >3.22, p2-tailed < 0.01). From left to right: left sagittal, top, and coronal views. The blue color of the wire indicates functional connections with reductions in values of lagged phase synchronization (LPS) from baseline to the end of stimulation in patients treated with hf-tRNS compared to sham group. Please, refer to Table S3 in the Supplementary materials for statistical details of the changes in functional connectivity between 84 regions positioning the center in BAs.

3. Supplementary tables

Table S1. A CONSORT checklist of this clinical trial.

CONSORT 2010 checklist of information to include when reporting a randomised trial

Section/Topic	Item No	Checklist item	Reported on page No
**Title and abstract**			
	1a	Identification as a randomised trial in the title	1
	1b	Structured summary of trial design, methods, results, and conclusions (for specific guidance see CONSORT for abstracts)	1
**Introduction**			
Background and objectives	2a	Scientific background and explanation of rationale	2-3
	2b	Specific objectives or hypotheses	3
**Methods**			
Trial design	3a	Description of trial design (such as parallel, factorial) including allocation ratio	3-4
	3b	Important changes to methods after trial commencement (such as eligibility criteria), with reasons	Not applicable
Participants	4a	Eligibility criteria for participants	3
	4b	Settings and locations where the data were collected	3
Interventions	5	The interventions for each group with sufficient details to allow replication, including how and when they were actually administered	3-5
Outcomes	6a	Completely defined pre-specified primary and secondary outcome measures, including how and when they were assessed	4
	6b	Any changes to trial outcomes after the trial commenced, with reasons	Not applicable
Sample size	7a	How sample size was determined	Supplementar y materials
	7b	When applicable, explanation of any interim analyses and stopping guidelines	Not applicable
Randomisation:			
Sequence generation	8a	Method used to generate the random allocation sequence	Supplementar y materials
	8b	Type of randomisation; details of any restriction (such as blocking and block size)	Supplementar y materials

Allocation concealment mechanism	9	Mechanism used to implement the random allocation sequence (such as sequentially numbered containers), describing any steps taken to conceal the sequence until interventions were assigned	supplementary materials
Implementation	10	Who generated the random allocation sequence, who enrolled participants, and who assigned participants to interventions	supplementary materials
Blinding	11a	If done, who was blinded after assignment to interventions (for example, participants, care providers, those assessing outcomes) and how	4
	11b	If relevant, description of the similarity of interventions	Not applicable
Statistical methods	12a	Statistical methods used to compare groups for primary and secondary outcomes	7
	12b	Methods for additional analyses, such as subgroup analyses and adjusted analyses	7
**Results**			
Participant flow (a diagram is strongly recommended)	13a	For each group, the numbers of participants who were randomly assigned, received intended treatment, and were analysed for the primary outcome	3-4
	13b	For each group, losses and exclusions after randomisation, together with reasons	Supplementary materials
Recruitment	14a	Dates defining the periods of recruitment and follow-up	4
	14b	Why the trial ended or was stopped	3
Baseline data	15	A table showing baseline demographic and clinical characteristics for each group	3
Numbers analysed	16	For each group, number of participants (denominator) included in each analysis and whether the analysis was by original assigned groups	3-4
Outcomes and estimation	17a	For each primary and secondary outcome, results for each group, and the estimated effect size and its precision (such as 95% confidence interval)	Not applicable
	17b	For binary outcomes, presentation of both absolute and relative effect sizes is recommended	Not applicable
Ancillary analyses	18	Results of any other analyses performed, including subgroup analyses and adjusted analyses, distinguishing pre-specified from exploratory	Not applicable
Harms	19	All important harms or unintended effects in each group (for specific guidance see CONSORT for harms)	Not applicable
**Discussion**			
Limitations	20	Trial limitations, addressing sources of potential bias, imprecision, and, if relevant, multiplicity of analyses	10
Generalisability	21	Generalisability (external validity, applicability) of the trial findings	10
Interpretation	22	Interpretation consistent with results, balancing benefits and harms, and considering other relevant evidence	Not applicable
**Other information**			
Registration	23	Registration number and name of trial registry	3
Protocol	24	Where the full trial protocol can be accessed, if available	3-4
Funding	25	Sources of funding and other support (such as supply of drugs), role of funders	11

Table S2. Associations between the changes in gamma-band EEG source-based large-scale functional network connectivity and the improvement in negative symptoms among participants treated with hf-tRNS when antipsychotic medication dose (in chlorpromazine equivalents) was controlled.

Variables	PANSS-FSNS score change from baseline to shortly after 10-session hf-tRNS		PANSS-FSNS score change from baseline to one-week follow-up		PANSS-FSNS score change from baseline to one-month follow-up	
	r	*p*	*r*	*p*	*r*	*p*
**Within-DMN gamma-band LPS changes from baseline to the end of stimulation**						
R_SFG - L_SFG	0.11	0.68	0.70	0.80	0.07	0.81
MFG - PC	-0.05	0.87	-0.14	0.59	-0.16	0.56
MFG - L_AG	-0.09	0.74	-0.23	0.40	-0.24	0.37
MFG - L_PHG	0.03	0.91	-0.11	0.69	-0.10	0.72
**Within-FPN gamma-band LPS changes from baseline to the end of stimulation**						
R_FP - L_MTG	-0.15	0.58	-0.33	0.21	-0.31	0.24
CG - L_SMG	-0.28	0.29	-0.48	0.06	-0.49	0.055

Abbreviations: PANSS, Positive and Negative Syndrome Scale; FSNS, Factor Score for Negative Symptoms; hf-tRNS, high-frequency transcranial random noise stimulation; DMN, default mode network; LPS, lagged phase synchronization; FPN, fronto-parietal network; R, right; L, left; SFG, superior frontal gyrus; MFG, medial frontal gyrus; PC, posterior cingulate; AG, angular gyrus; PHG, parahippocampal gyrus; FP, frontal pole; MTG, middle temporal gyrus; CG, cingulate gyrus; SMG, supramarginal gyrus.

Table S3. Results of statistical details of the changes in whole-brain functional connectivity between 84 regions positioning the center of Brodmann areas (BAs). The t values that are highlighted in fluorescence yellow and green indicate they exceed the threshold of 2.825 ($p_{2\text{-tailed}} < 0.05$) and 3.22 ($p_{2\text{-tailed}} < 0.01$), respectively.

Table S3. Xlsx

Table S4. The statistically significant changes in whole-brain functional connectivity between 84 regions positioning the center of Brodmann areas (BAs) with the t values exceeding the threshold of 3.22 (p2-tailed < 0.01).

**Functional connectivity**<br><br>The ROIs at the centroid of Brodmann Areas (MNI X, Y, Z coordinates)	**t value**
Middle Frontal Gyrus_R (30, 30, 35)_Cuneus_L (-25, -75, 10)	-3.27
Middle Frontal Gyrus_R (30, 30, 35)_Posterior Cingulate_L (-5, -50, 5)	-3.49
Middle Frontal Gyrus_R (30, 30, 35)_Posterior Cingulate_L (-15, -60, 5)	-3.32
Lingual Gyrus_R (10, -90, 0)_Anterior Cingulate_L (-5, 20, 20)	-3.33
Lingual Gyrus_R (15, -80, 0)_Anterior Cingulate_L (-5, 20, 20)	-3.41
Cingulate Gyrus_R (5, 0, 35)_Paracentral Lobule_R (15, -45, 60)	-3.32
Anterior Cingulate_R (0, 20, 20)_Lingual Gyrus_R (10, -90, 0)	-3.24
Anterior Cingulate_R (0, 20, 20)_Lingual Gyrus_R (15, -80, 0)	-3.28
Superior Temporal Gyrus_R (40, 15, -30)_Posterior Cingulate_L (-15, -60, 5)	-3.29

Notes: Coordinates are in MNI space. L=Left hemisphere seed; R=Right hemisphere seed; X=left (−) to right (+); Y=posterior (−) to anterior (+); Z=inferior (−) to superior (+); R – right; L – left; ROIs, regions of interest.

References:

1. Schlegelmilch, F., Markert, S., Berkes, S. & Schellhorn, K. (2004). Online ocular artifact removal for dc-EEG-signals: Estimation of dc-level. Biomedizinesche Technik, Ergänzungsband 2., Band, 49, 340-341.
